# Depression and stress regarding future career among university students during COVID-19 pandemic

**DOI:** 10.1371/journal.pone.0266686

**Published:** 2022-04-12

**Authors:** Upama Chowdhury, Md. Ahosan Habib Suvro, Syed M. D. Farhan, Md Jamal Uddin

**Affiliations:** Department of Statistics, Shahjalal University of Science & Technology, Sylhet, Bangladesh; National Cheng Kung University College of Medicine, TAIWAN

## Abstract

**Introduction:**

Students generally struggle to build a good career after their graduation in developing countries like Bangladesh. Due to the COVID-19 pandemic, such struggle increased and faced with stress and depression. We aimed to inquire about the amplitude of depression and stress among university students during the COVID-19 pandemic regarding their future careers and to identify the factors associated with this depression and stress.

**Methods:**

A total of 516 students at various universities participated in this cross-sectional survey. From October 2020 to February 2021, data was collected through an online survey. An e-questionnaire with socio-demographic, Patient Health Questionnaire (PHQ-9) and Perceived Stress Scale (PSS-10) questions was created using Google Forms and distributed via Facebook, WhatsApp, and other social media platforms. The universities included into the sample were selected randomly from eight divisions of Bangladesh. Descriptive statistics and Pearson chi-square tests were carried out and the association between the risk factors and the outcome (e.g. depression and stress) was assessed by the odds ratio (OR) including 95% confidence interval (CI) obtained from the binary logistic regression model.

**Results:**

Among 516 participants, 380 (73.6%) were male and 136 (26.4%) were female. Around 414 (80.2%) participants had mild to severe depression and 399 (77.3%) reported having low to moderately perceived stress. Female students were 2.1 (95% CI: 1.21–3.76) times more depressed and 3.6 (95% CI: 1.87–6.76) times more stressed than the counterpart. Students, who think delaying graduation due to COVID-19 will reduce the chance of getting a job, were 1.72 (95% CI: 1.07–2.76) times more depressed. Respondents, whose department offers any internship were 36% less depressed (p = 0.053), while skilled students were 46% less stressed though it was not statistically significant (p = 0.43).

**Conclusion:**

According to our findings, there is an increasing prevalence of depression and stress among students, particularly among female students and those who do not receive job-related facilities from their institutions or who are unskilled. Universities can provide mental health programs and strive to have enough space for students to participate in internships. In addition, the government and educational institutions should work together to address the growing challenge.

## Introduction

Stress is an emotional force that creates bodily or mental tension, according to physiological or biological contexts. It may be caused by an upsetting, frustrated, or nervous experience or feeling [1]. Although acute or short-term stress can be beneficial, chronic stress, which lasts for a longer period, is extremely detrimental to the body and mind, causing high blood pressure, weight gain, depression, and even heart disease. Chronic stress can even lead to suicidal ideation [2]. While stress and depression have several parallels, depression is a mental disorder characterized by persistent unhappiness and a lack of desire. Clinical depression, also known as major depressive disorder, affects a person’s thoughts, perceptions, and behavior and can result in a number of emotional and physical problems [3].

A novel coronavirus was discovered in December 2019 [4], and the virus’s dissemination soon became a global health threat. After the outbreak of COVID-19 which was affecting the lives of people all over the world, the World Health Organization (WHO) announced that it is a global pandemic in the second week of March 2020 [5]. Aside from the physical impacts, it has had a detrimental impact on the mental health of the students [6]. During the pandemic, a study in Iran found that 38% of students had anxiety issues, and 28% had mild to severe depression [7]. Another study discovered that during COVID-19, undergraduate students showed higher mean perceived stress and anxiety compared to postgraduate students. That is, during this global pandemic, stress and anxiety increased among nearly graduate students [8]. Moreover, the COVID-19 has caused depressive and psychological distress in individuals, particularly students who are looking for work and have future career plans [9–11]. Fear of COVID-19 was positively related to job insecurity and future career attributes, which increases depression and psychological problems [9, 11]. It is delineated in different studies that being faced with a pandemic is extremely stressful, and those who have experienced such situations are more sad [12–14]. Furthermore, students are scared and nervous during pandemics, and psychological illnesses are frequent, as a link was established between lengthier quarantine durations and stress and depression among persons during the severe acute respiratory syndrome (SARS) epidemic [15, 16].

Depression and stress are also considered to be one of the major causes of suicide among university students [17, 18]. While university students consistently suffer from depression and stress due to the factor like academic success or academic result [19–21], the COVID-19 outbreak has increased the depression and stress rate among university students alarmingly [22]. In a recent Bangladeshi study, it has been found that COVID-19 has made fear for future career among the university students and COVID-19 fear is positively correlated with future career anxiety and the relation is highly significant [23]. Given the unusual circumstances, investigating the psychosocial experiences of university students regarding the constant pressure they are facing about their future careers in Bangladesh, particularly during the outbreak of the COVID-19 pandemic, is convincing. Additionally, students are having mental and financial pressure as many people became jobless, homeless, and lost their companies. University students are already under constant stress due to variables such as academic demands, financial crisis, delay graduation, and career choices [24].

Bangladesh had the first case of COVID-19 on March 8, 2020, and to stifle the transmission government placed a complete lockdown on March 26, 2020 [25–27]. During COVID-19, several studies are conducted in Bangladesh based on the depression and anxiety of the students. For example, after the lockdown, a study on Bangladeshi students showed that more than 80% students have mild to severe depression where male students have higher depressive symptoms [28, 29]. Also, it was observed that among the underlying factors playing behind this mental breakdown among the young adults, unemployment, financial instability and uncertainty of job were determined as some major culprits [30, 31]. However, this studies lack information on the issue of depression and/or stress among university students during the COVID-19 pandemic in terms of their future careers. Therefore, we aimed to investigate the depression and stress regarding future career among university students during COVID-19 pandemic and identify the factors causing depression and stress.

## Methodology

### Study design, setting and participants

The data for this cross-sectional study was collected from the students of randomly selected different public and private universities of all 8 divisions of Bangladesh between October 2020 and February 2021. An easy and simple e-questionnaire with socio-demographic, Patient Health Questionnaire (PHQ-9) and Perceived Stress Scale (PSS-10) questions was created using Google Forms and distributed via Facebook, WhatsApp, and other social media platforms. The reliability of this questionnaire was also reasonable (Cronbach’s alpha [α] = 0.78). A pilot study was conducted among 35 students to determine the feasibility and clarity of the study. Later they were excluded from the data analysis. Before the final study was conducted, the questionnaire went through some refinements as required. After this the students of 3^rd^ year, 4^th^ year and Masters from 62 renowned universities and colleges were selected as they were close enough to finish their graduation and going to enter the job market. And also in previous studies, it has been noticed that the evidence of depression and stress was higher among the older students [31]. The minimum sample size for this study was determined around 504 by using the formula of sample size for cross-sectional study for qualitative variable where P = 0.7 was determined by the pilot survey and previous studies and d = 0.04.

### Ethical approval

The ethics application has been approved by the Biostatistics research group, Department of Statistics, Shahjalal University of Science and Technology, Sylhet-3114, Bangladesh (*no*. *sta/2020/6/upama/01*). All procedures were carried out in line with the institutional and/or national research committee’s ethical requirements, as well as the 1964 Helsinki statement and its subsequent revisions or comparable ethical standards.

### Measurements

The Perceived Stress Scale, PSS-10 [32] questionnaire was used to assess the severity of stress, while the Patient Health Questionnaire, PHQ-9 [33] questionnaire was used to evaluate depression levels. Socio-demographic information section contains some personal information about the participants including gender, family income status, age, study year, living area, household income, extracurricular activities, managing study cost, living with family, financially support himself, father’s occupation, mother’s occupation. The information related to the respondents like COVID-19 hindered future career planning, startup plan, having skills, internship offered by the department, any job company closed due to COVID-19, skill development course, perspective of the informants about different job sectors and job opportunities are covered in this section.

The simple but effective tool, the PHQ-9 questionnaire, was used to evaluate depression among the students. It is highly effective and reliable in inspecting the intensity of depressive states among university students [34]. In the PHQ-9 module, each of the 9 DSM-IV criteria is scored as “0” (not at all) to “3” (nearly every day) [33]. The intervals 0–4, 5–9, 10–14, 15–19, 20 to above of PHQ-9 questionnaire represents minimal, mild, moderate, moderately severe, severe depression levels respectively [33]. For simplicity, these five categories have been transferred into two categories by diving them as ‘no’ = no to minimal depression and ‘yes’ = mild to severe depression. Stress was determined by using the PSS-10 Questionnaire. PSS-10 is an easy and reliable way to measure stress [32]. On a 5-point Likert scale, respondents were asked how often they felt or thought about each of the 10 things in the previous month (0 = never, 1 = practically never, 2 = sometimes, 3 = pretty often, and 4 = very often) [35]. The levels of stress for the study were categorized as low/no stress = 0–13, moderate stress = 14–26, high perceived stress = 27–40 [35].

### Statistical analysis

For describing, comparing, and summarizing the socio-demographic and future career-related information of the respondents, descriptive statistics and Pearson chi-square test were carried out. All the categorical variables were compared with depression and stress while the significant factors of univariate and bivariate linear regression were entered into the binary logistic regression model. In contrast to general regression, which uses parameters to minimize the sum of squared errors, logistic regression uses parameters to maximize the likelihood function of the observed samples. The forward selection was used as the mode of entering variables in the model. We first fitted univariate model to estimate the effect of different variables on the outcome variables. In the multivariable analysis we entered different independent variables to measure their influence on the outcome variables (depression and stress) which were categorized into two divisions as Yes = 0 and No = 1. The association between the risk factors and the outcome (e.g. depression and stress) was assessed by the odds ratio (OR) including 95% confidence interval (CI) obtained from the binary logistic regression model. All the analysis of this study was carried out using the SPSS version 25.0.

## Results

### Basic information of respondents

**Table 1** shows the descriptive information of the study in which 380 (73.6%) of the respondents were male and 136 (26.4%) were female. A big number of students (70.3%) need family support to manage their study costs and 55% of students financially support themselves. Around 75% of the students live with their families. It also shows that females were significantly more depressed (88.2% vs. 77.4%; p = 0.006) and stressed (90.4% vs. 72.4%; p <0.001) compared to male. The variable managing study cost is significantly associated with stress (p < 0.001) respectively. The variable family income status is also significantly associated with stress (p = 0.015). The respondents who were worried about getting infected by COVID-19 were significantly depressed (p = 0.013) and stressed (p = 0.009).

**Table 1:** Distribution of the socio demographic variables by depression and stress.

*Variables*	*n(%)*	*Depression*				*Stress*			
		Yes	No	*χ* ^2^	p-values	Yes	No	*χ* ^2^	p-values
		414(80.2%)	102(19.8%)			(399; 77.3%)	(117;22.7%)		
**Gender**				7.457	0.006*			18.118	<0.001**
Male	380(73.6)	294(77.4)	86(22.4)			276(72.6)	104(27.4)		
Female	136(26.4)	120(88.2)	16(11.8)			123(90.4)	13(9.6)		
**Living Area**				0.694	0.405			0.048	0.826
Urban	322(62.4)	262(81.4)	60(18.6)			250(77.6)	72(22.4)		
Rural	194(37.6)	152(78.4)	42(21.6)			149(76.8)	45(23.2)		
**Study Year**				0.751	0.687			7.455	0.024*
3^rd^ Year	210(40.7)	171(81.4)	39(18.6)			175(83.3)	35(16.7)		
4^th^ Year	265(51.4)	212(80)	53(20)			195(73.6)	70(26.4)		
MS	41(7.9)	31(75.6)	10(24.4)			29(70.7)	12(29.3)		
**Age**				1.56	0.458			4.397	0.111
20–22	257(49.8)	211(82.1)	46(17.9)			208(80.9)	49(19.1)		
23–25	251(48.6)	196(78.1)	55(21.9%)			186(74.1)	65(25.9)		
>25	8(1.6)					5(62.5)	3(37.5)		
**Managing Study Cost**				4.695	0.096			19.334	<0.001**
Family Support	363(70.3)	284(78.2)	79(21.8)			262(72.2)	101(27.8)		
Tuition	123(23.8)	107(87)	16(13)			112(91.1)	11(8.9)		
Others	30(5.8)	23(76.7)	7(23.3)			25(83.3)	5(16.7)		
**Income Status**				5.786	0.055			8.354	0.015*
Low Income	74(14.3)	63(85.1)	11(14.9)			62(83.8)	12(16.2)		
Medium Income	342(66.3)	279(81.6)	63(18.4)			270(78.9)	72(21.1)		
High Income	100(19.4)	72(72)	28(28)			67(67.0)	33(33.0)		
**Financially Support Himself**				1.304	0.253			0.007	0.933
Yes	284(55.0)	233(82)	51(18)			220(77.5)	64(22.5)		
No	232(45.0)	181(78)	51(22)			179(77.2)	53(22.8)		
**Living with Family**				2.753	0.097			1.33	0.249
Yes	387(75.0)	317(81.9)	70(18.1)			304(78.6)	83(21.4)		
No	129(25.0)	97(75.2)	32(24.8)			95(73.6)	34(26.4)		
**Extracurricular Activities**				1.096	0.295			2.969	0.085
Yes	295(57.2)	232(78.6)	63(21.4)			220(74.6)	75(25.4)		
No	221(42.8)	182(82.4)	39(17.6)			179(81.0)	42(19.0)		
**Worried about infected by COVID-19**				6.164	0.013*			6.779	0.009*
Yes	379(73.4)	314(82.8)	65(17.2)			304(80.2)	75(19.8)		
No	137(26.6)	100(73)	37(27)			95(69.3)	42(30.7)		

*significant at p < 0.05;

**significant at p < 0.001.

### Prevalence of depression and stress

**Fig 1:**
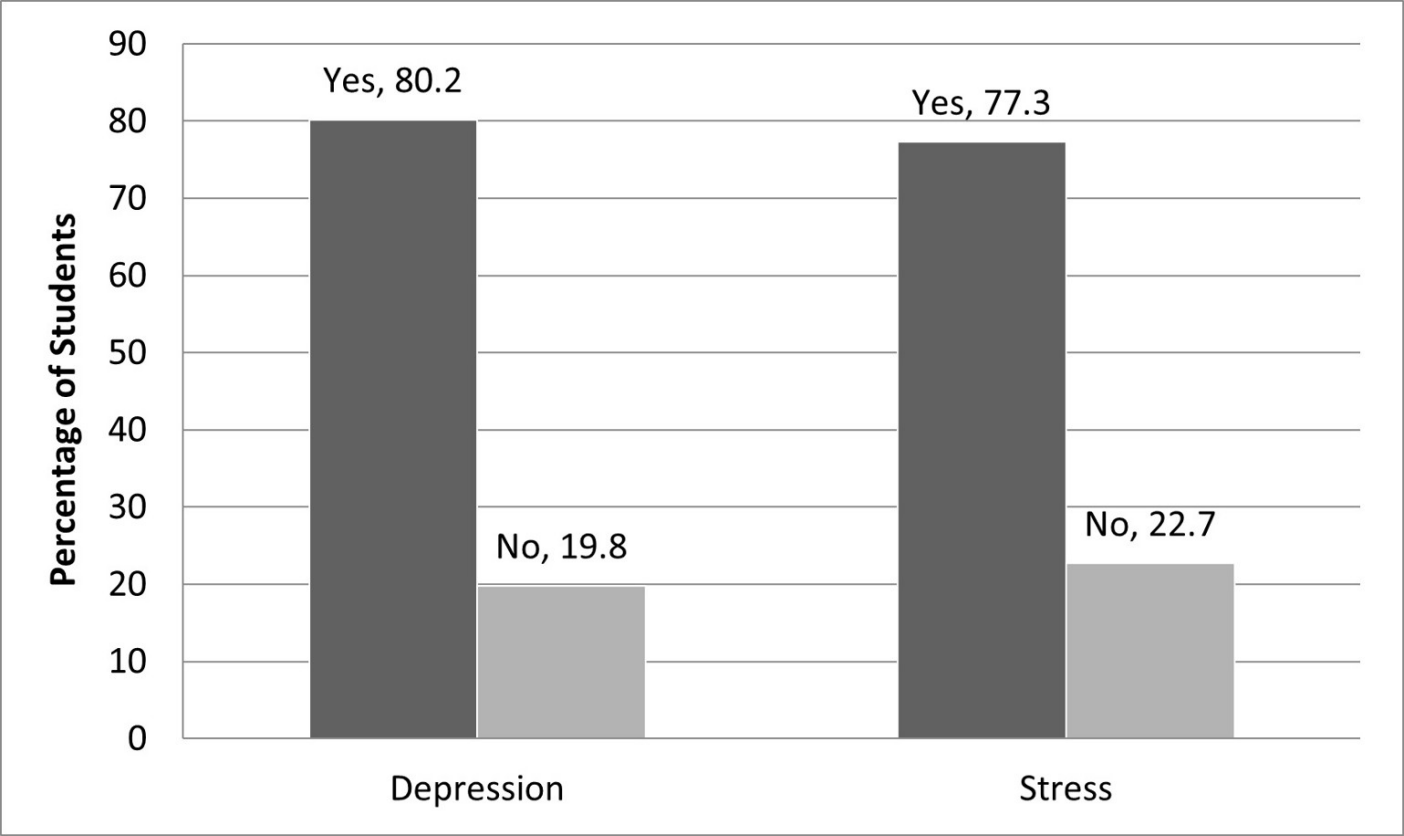
Distribution of students in the depression and stress groups.

**Fig 1** shows the prevalence of depression and stress among the participants. Mild to severe depressive symptoms were found in 414 (80.2%) participants and among 399 (77.3%) moderate to moderately severe stress was found (Fig 1). Female students (88.3%) showed higher severity in depression than the male (77.4%). Out of 9 schools of different universities in Bangladesh, students of Physical Science and Life Sciences showed the most severity (82.6% and 81% showed mild to severe depression). The prevalence of depression is higher for the students who live with their families (81.9%) than the counterpart. The students whose departments do not offer any course for skill development (81.7%) and do not offer any internship (81.4%) showed higher depressive symptoms. In the case of stress female students (90.5%) showed higher stress than the male students (72.6%). Like depression, the school of Physical Science and Life Science students showed higher stress and the students living with family had higher stress (78.5%). The students whose departments do not offer any course for skill development or any internship were found to have higher stress symptoms (**Table 2**).

**Table 2:** Results of Patient Health Questionnaire (PHQ-9) and Perceived Stress Scale (PSS-10).

*Variables*	*Depression*					*Stress*		
	None to minimal	Mild	Moderate	Moderately Severe	Severe	Low Stress	Moderate Stress	Moderately Perceived Stress
**Gender**								
Male	86(22.6)	114(30.0)	78(20.5)	80(21.1)	22(5.8)	104(27.4)	273(71.8)	3(0.8)
Female	16(11.8)	30(22.1)	30(22.1)	43(31.6)	17(12.5)	13(9.6)	118(86.8)	5(3.7)
**Schools**								
Agriculture	6(33.3)	4(22.2)	2(11.1)	4(22.2)	2(11.1)	7(38.9)	11(61.1)	0(0.0)
Applied Sciences	43(21.2)	53(26.1)	53(26.1)	41(20.2)	13(6.4)	43(21.2)	159(78.3)	1(0.5)
Business Administration	8(16.3)	11(22.4)	13(26.5)	16(32.7)	1(2.0)	13(26.5)	34(69.4)	2(4.1)
Law	1(20.0)	1(20.0)	0(0.0)	1(20.0)	2(40.0)	1(20.0)	3(60.0)	1(20.0)
Life Sciences	4(19.0)	7(33.3)	3(14.3)	6(28.6)	1(4.8)	3(14.3)	17(81.0)	1(4.8)
Medical	2(25.0)	3(37.5)	3(37.5)	0(0.0)	0(0.0)	3(37.5)	5(62.5)	0(0.0)
Pharmacy	0(0.0)	1(25.0)	2(50.0)	0(0.0)	1(25.0)	1(25.0)	3(75.0)	0(0.0)
Physical Sciences	21(17.4)	42(34.7)	19(15.7)	27(22.3)	12(9.9)	25(20.7)	93(76.9)	3(2.5)
Social Sciences	17(19.5)	22(25.3)	13(14.9)	28(32.2)	7(8.0)	21(24.1)	66(75.9)	0(0.0)
**Study Year**								
3^rd^ Year	39(18.6)	57(27.1)	45(21.4)	50(23.8)	19(9.0)	35(16.7)	169(80.5)	6(2.9)
4^th^ Year	53(20.0)	77(29.1)	54(20.4)	63(23.8)	18(6.8)	70(26.4)	194(73.2)	1(0.4)
Masters	10(24.4)	10(24.4)	9(22.0)	10(24.4)	2(4.9)	12(29.3)	28(68.3)	1(2.4)
**Living place**								
Urban	60(18.6)	92(28.6)	71(22.0)	74(23.0)	25(7.8)	72(22.4)	242(75.2)	8(2.5)
Rural	42(21.6)	52(26.8)	37(19.1)	49(25.3)	14(7.2)	45(23.2)	149(76.8)	0(0.0)
**Living with family**								
Yes	70(18.1)	106(27.4)	82(21.2)	98(25.3)	31(8.0)	83(21.4)	297(76.7)	7(1.8)
No	32(24.8)	38(29.5)	26(20.2)	25(19.4)	8(6.2)	34(26.4)	94(72.9)	1(0.8)
**Extracurricular activities**								
Yes	63(21.4)	81(27.5)	63(21.4)	69(23.4)	19(6.4)	75(25.4)	215(72.9)	5(1.7)
No	39(17.6)	63(28.5)	45(20.4)	54(24.4)	20(9.0)	42(19.0)	176(79.6)	3(1.4)
**Skill development course by department**								
Yes	42(22.2)	56(29.6)	44(23.3)	38(20.1)	9(4.8)	54(19.3)	132(69.8)	3(1.6)
No	60(18.3)	88(26.9)	64(19.6)	85(26.0)	30(9.2)	63(19.3)	259(79.2)	5(1.5)
**Internship offered by department**								
Yes	35(22.0)	51(32.1)	36(22.6)	30(18.9)	7(4.4)	45(28.3)	111(69.8)	3(1.9)
No	67(18.8)	93(26.1)	72(20.2)	93(26.1)	32(9.0)	72(20.2)	280(78.4)	5(1.4)

### Future-career related information of study participants

**Table 3** shows that those who think COVID-19 hindered their future plan were more depressed (82.3% vs. 71.7%; p = 0.018) and stressed (79.4% vs. 68.7%; p = 0.022) than their counterpart. For the lack of the scope of communicating with the students of the companies, it caused highly significant depression (p < 0.001) also significant stress (p = 0.041). Those whose departments do not offer internship were significantly stressed (79.8% vs.71.7%; p = 0.042) than the students whose department offer internship. Moreover, depression and stress were significantly associated with skill for desired job (p = 0.021 & p = 0.001 respectively) and stress had a significant association with the preparation for other job sectors (p = 0.017 respectively).

**Table 3:** Distribution of the future career related variables by depression and stress.

*Variables*	*n(%)*	*Depression*				*Stress*			
		Yes	No	*χ* ^2^	p-values	Yes	No	*χ* ^2^	p-values
		(414; 80.2%)	(102; 19.8%)			(399;77.3%)	(117;22.7%)		
**COVID-19 is hindering future plan**									
Yes	417(80.8)	343(82.3)	74(17.7)	5.601	0.018*	331(79.4)	86(20.6)	5.214	0.022*
No	99(19.2)	71(71.7)	28(28.3)			68(68.7)	31(31.3)		
**Delaying graduation can decrease the chance of getting job**									
Yes	388(75.2)	320(82.5)	68(17.5)	4.956	0.026*	306(78.9)	82(21.1)	2.117	0.146
No	128(24.8)	94(73.4)	34(26.6)			93(72.7)	35(27.3)		
**Interested job company had been closed due to COVID-19**									
Yes	159(30.8)	134(84.3)	25(15.7)	2.370	0.124	132(83.0)	27(17.0)	4.249	0.039*
No	357(69.2)	280(78.4)	134(84.3)			267(74.8)	90(25.2)		
**Company can’t reach fresher due to pandemic**									
Yes	448(86.8)	376(83.9)	72(16.1)	29.281	<0.001**	353(78.8)	95(21.2)	4.184	0.041*
No	68(13.2)	38(55.9)	30(44.1)			46(67.6)	22(32.4)		
**Course by department for job skill**									
Yes	189(36.6)	147(77.8)	42(22.2)	1.133	0.287	135(71.4)	54(28.6)	5.915	0.015*
No	327(63.4)	267(81.7)	60(18.3)			264(80.7)	63(19.3)		
**Internship offered by department**									
Yes	159(30.8)	124(78)	35(22)	0.730	0.393	114(71.7)	45(28.3)	4.151	0.042*
No	357(69.2)	290(81.2)	67(18.8)			285(79.8)	72(20.2)		
**Department that offers internship has better chance at getting job**									
Yes	444(86.0)	360(81.1)	84(18.9)	1.445	0.229	350(78.8)	94(21.2)	4.101	0.043*
No	72(14.0)	54(75)	18(25)			49(68.1)	23(31.9)		
**Skilled enough for desired job**									
Yes	220(42.6)	165(75)	55(25)	6.621	0.01*	154(70.0)	66(30.0)	11.73	0.001*
No	296(57.4)	249(84.1)	47(15.9)			245(82.8)	51(17.2)		
**Prepared for other job sectors**									
Yes	228(44.2)	175(76.8)	53(23.2)	3.116	0.078	165(72.4)	63(27.6)	5.725	0.017*
No	288(55.8)	239(83)	49(17)			234(81.3)	54(18.8)		
**Plan for startup**									
Yes	279(54.1)	223(79.9)	56(20.1)	0.035	0.851	219(78.5)	60(21.5)	0.473	0.491
No	237(45.9)	191(80.6)	46(19.4)			180(75.9)	57(24.1)		

*significant at p < 0.05;

**significant at p < 0.001.

### Factors related to depression and stress

Logistic regression in **Table 4** shows that female students were 2.1 times (95% CI: 1.21–3.76) more depressed and 3.6 times (CI: 1.87–6.76) more stressed than the male students. Those students who manage their study cost by doing tuition were 2.5 times (CI: 1.38–4.67) more likely to be depressed and 4.2 times (CI: 2.10–8.36) more stressed than the students who have family support. Students who manage study costs by other ways like a scholarship or online income were 2.9 times (CI: 1.024–8.064) more stressed. Students who were living with their families had 1.7 times (CI: 1.07–2.82) more depression than those who were not living with family. Among the respondents whose departments offer any internship are 36% less depressed (p = 0.053) than their counterparts.

**Table 4:** Odds ratio (OR) from logistic regression models for the depression and stress.

Variables	*Depression*				*Stress*			
	*Estimates*	OR	95% CI	p-value	*Estimates*	OR	95% CI	p-values
**Gender**								
Male	*Reference*				*Reference*			
Female	0.759	2.13	1.21–3.76	0.009*	1.271	3.56	1.87–6.76	<0.001**
**Place of residence**								
Urban	*Reference*				*Reference*			
Rural	-0.151	0.86	0.55–1.34	0.507	-0.032	0.96	0.61–1.53	<0.001**
**Manage study cost**								
Family Support	*Reference*				*Reference*			
Tuition	0.935	2.54	1.38–4.67	0.003*	1.435	4.2	2.10–8.36	<0.001**
Others	0.504	1.65	0.65–4.19	0.288	1.055	2.87	1.02–8.06	0.045*
**Living with family**								
No	*Reference*				*Reference*			
Yes	0.554	1.74	1.07–2.82	0.025*	0.273	1.31	0.79–2.17	0.291
**Delaying graduation can decrease the chance of getting job**								
No	*Reference*				*Reference*			
Yes	0.547	1.72	1.07–2.76	0.023*	0.2	1.22	0.74–2.00	0.428
**Skilled for desires job**								
No	*Reference*				*Reference*			
Yes	-0.341	0.71	0.45–1.10	0.128	-0.607	0.54	0.34–0.85	0.428
**Internship offered by department**								
No	*Reference*				*Reference*			
Yes	-0.442	0.64	0.41–1.00	0.053	-0.332	0.71	0.45–1.13	0.157
**Family pressuring for financial support**								
No	*Reference*				*Reference*			
Yes	0.504	1.65	0.99–2.74	0.052	0.372	1.45	0.87–2.41	0.157
**Startup plan**								
No	*Reference*				*Reference*			
Yes	0.417	1.51	0.98–2.34	0.06	0.139	1.15	0.73–1.79	0.541

CI, confidence Interval; OR, odds ratio;

*significant at p < 0.05;

**significant at p < 0.001.

## Discussion

This study intends to assess the prevalence of depression and stress among university students in Bangladesh during the COVID-19 pandemic regarding their future careers and to identify the factors related to this depression and stress. We found that gender, managing study cost by own, living with family during the pandemic, delaying graduation, less skilled, no internship opportunity, pressure by the family for financial support and start-up plan are notably associated with depression and stress among the students considering their future career; some of which shows consistency with previous studies on university students in other developing countries [29, 30].

The findings of this study indicate that a large segment of university students are dealing with depression (76.4%) and stress (77.3%) while a previous study among the students of an university in Egypt showed the prevalence of stress and depression was 62.4% and 60.8% respectively [36]. Also a study among medical students in Bangladesh showed 52.2% of students were suffering from depression [37] and another study found that 54.3% students were dealing with depression while 59% students were suffering from stress [38]. A study among the medical students of India also showed that more than fifty percent of the students were affected by depression (51.3%) and stress (53%) [39]. This study indicates a sudden and unparalleled change in both depression and stress among university students after analyzing previous studies.

This study suggests that the probability of getting a suitable job was negatively related to the time length of graduation. Nowadays most of the private company prefers youths as they are more innovative, and they can adapt easily. Moreover, the executive or chief positions of a company need an experienced employee while delaying graduation will affect their time to get experience. A previous study showed that older graduated students are more depressed and stressed than the fresh graduates for the Bangladesh Civil Service (BCS) exam [40]. As the age limit for applying for a government job is thirty [41] for delaying graduation the age limit will be lesser for the fresh graduates for applying in any government job which is causing depression and stress among the university students.

The findings also showed that internship was also a key factor for understanding the increased depressive symptoms and stress among university students. An internship can not only enhance one’s professional experience but also increase the opportunity to get a better job [42]. An internship can also be considered as the sample test by the respective company for the elongated type of works. In a survey, it has been found that interns who were more into self-promotion and ingratiation are likely to be hired by the host company [43]. In this study, it has been found that the students of those departments that offer internships after graduation are likely to show less depressive and stress symptoms than the counterpart.

While start-up or working as an entrepreneur is a blooming career path according to the youths, the COVID-19 pandemic has shown destructive consequences on this sector. According to a survey conducted by a business consultancy firm in Bangladesh during the pandemic, 24% of startups have been forced to cease their operations [44]. As in recent years, startups in Bangladesh are growing consistently [45], thus many university students are also regarding this sector as their future career. Though startups are considered to be popular and tend to be a shoestring operation, without experience the effectiveness and survival probability of a start-up team in long terms are very low [46]. And to gain better experience it is undeniable to work with experienced persons or team or under a professional which can be gained by involving in internships.

This study points high perceived depression and stress among female students. In some previous studies, it was found that the symptoms of depression and stress among female university students are higher than the male university students [47, 48]. Delaying graduation can be a factor for depression as we have found a significant high depressive symptom as well as stress for those students. Their chances for marriage after building a bright career will be demolished for the delay. Some research tells us that marriage collapses the probability of being employed of a woman [49], also women having offspring after marriage decreases the scope of doing any job [50] as they have to take care of the newborn. From that mindset, a woman can have severe depression and stress thinking that she will or may not get a chance to be employed after marriage.

Most Bangladeshi university students are involved in different types of part-time jobs like tuition, freelancing, and different online-based works. Among all the part-time jobs for students, private tuition is the topmost part-time job and a study on the private supplementary tuition among the students of Bangladesh has suggested that more than 30% of the tutors are university students (31.3%) [51]. This study also suggests that among the students, 23.8% of students finance their tuition fees, and more than 50% of students used to do something to financially support themselves and their family (55%). But as the stay-at-home strategy had been officially ordered to put on the action, the educational institutions had been closed. This also caused most of the students to lose their income sources as most of them used to live in a dormitory or around their respective campus and because of lockdown, they had to leave the campus. Hence sudden unemployment leads to financial insecurities which may cause mental health problems [52]. Additionally, in a study, it was found that in comparison to other nations, the negative impact of unemployment on mental health was greater in countries with a low level of economic growth, uneven income distributions, or poor unemployment protection systems [53].

According to the findings of the study, living with family is a major factor as those who are living with their family during the pandemic showed more mild-to-severe symptoms of depression. During the COVID-19 pandemic, many families lost their income sources as some company such as tourism and hotel business has been completely shut down or has been closed temporarily [54]. So, many families are facing a financial crisis and they are giving pressure on the students to get involved in the job industry for contributing financially. In previous studies, it was shown that a healthy and friendly environment of a family helps to reduce symptoms of depression and stress, while mental health was severely disturbed due to the negative environment of a family [55].

The study exhibits that the factor place of residence of the students has no significant relationship with both depression and stress which was also observed in a previous study conducted on Bangladeshi university students [22]. Other socio-demographic variables like age, study year, and extra-curricular activity were not significantly related to depression and stress respectively which shows similarity with a previous study on undergraduate students [56]. Meanwhile, some future career-related variables like ‘Interested job company had been closed due to COVID-19 and Skill development course offered by the department had also shown insignificant relation with depression and stress.

### Strengths and limitations

The strengths and drawbacks of this study can be composed of different aspects. An e-questionnaire that was used to collect the data was the main strength of the study. It generously helped to collect the information from the respondents. The respondents were able to respond spontaneously with the help of the questionnaire. As World Health Organization (WHO) recommended social distancing during the pandemic it was impossible to go door to door of the respondents, so contactless data collection came to possible with the help of the e-questionnaire. For the e-questionnaire, it needed no funds for collecting the data such as travel cost, volunteer cost which is also a great strength of the study. The sample size of the study is slightly small, which is a limitation of the study. The sample size could be a little bigger so that it could be more representative of the population. The study is a cross sectional study and the main limitation of this study is it cannot be measured the cause and effect. In this study self-report data collection was used that means responders gave their information of their own responsibility. So, there can be some errors or mistakes made by the respondents which is also a limitation for this study.

## Conclusions

Our study reveals that there is a high perceived growing depression and stress due to the COVID-19 pandemic among the students of Bangladesh for their future careers. Delaying graduation, lack of skills for getting a suitable job, startup plan, and getting no internship facilities from their institution is making a big contribution to the depression and stress. Government as well as the universities should collaborate to inhibit the situation by providing online mental health care programs and internships after graduation. Students should self-support themselves and should be self-enterprising, that they can erase their mental pressure and overcome depression and stress.
